# Phylogenetic Reconstructions Reveal the Circulation of a Novel Dengue Virus-1V Clade and the Persistence of a Dengue Virus-2 III Genotype in Northeast Brazil

**DOI:** 10.3390/v15051073

**Published:** 2023-04-28

**Authors:** Hegger Fritsch, Keldenn Moreno, Italo Andrade Barbosa Lima, Cleiton Silva Santos, Bernardo Gratival Gouvea Costa, Breno Lima de Almeida, Ronald Alves dos Santos, Marcos Vinicius Lima de Oliveira Francisco, Maria Paula Souza Sampaio, Maricelia Maia de Lima, Felicidade Mota Pereira, Vagner Fonseca, Stephane Tosta, Joilson Xavier, Carla de Oliveira, Talita Adelino, Arabela Leal e Silva de Mello, Tiago Gräf, Luiz Carlos Junior Alcantara, Marta Giovanetti, Isadora Cristina de Siqueira

**Affiliations:** 1 Instituto de Ciência Biológicas, Universidade Federal de Minas Gerais, Avenida Presidente Antônio Carlos, 6627, Belo Horizonte 31270-901, MG, Brazil; 2 Instituto Gonçalo Moniz, Fundação Oswaldo Cruz, Rua Waldemar Falcão, 121, Salvador 40296-710, BA, Brazil; 3 Secretaria Municipal de Saúde de Feira de Santana, Avenida João Durval Carneiro, s/n, Feira de Santana 44027-010, BA, Brazil; 4 Departamento de Saúde, Universidade Estadual de Feira de Santana, Avenida Transnordestina, s/n, Feira de Santana 44036-900, BA, Brazil; 5 Laboratório Central de Saúde Pública Prof Goncalo Moniz, Rua Waldemar Falcão, 123, Salvador 40295-010, BA, Brazil; 6 Organização Pan-Americana de Saúde/Organização Mundial de Saúde, Setor das Embaixadas, Lote 19, Avenida das Nações, Brasília 70-800400, SP, Brazil; 7 Laboratório de Flavivírus, Lnstituto Oswaldo Cruz/Fundação Oswaldo Cruz, Avenida Brasil, 4365, Rio de Janeiro 21040-900, RJ, Brazil; 8 Laboratório Central de Saúde Pública do Estado de Minas Gerais, Fundação Ezequiel Dias, Rua Conde Pereira Carneiro, 80, Belo Horizonte 30510-010, MG, Brazil; 9 Laboratório de Virologia Molecular, Instituto Carlos Chagas/Fiocruz-PR, Avenida Professor Algacyr Munhoz Mader, 3775, Curitiba 81310-020, PA, Brazil; 10 Instituto Rene Rachou, Fundação Oswaldo Cruz, Avenida Augusto de Lima, 1715, Belo Horizonte 30190-002, MG, Brazil; 11 Sciences and Technologies for Sustainable Development and One Health, University of Campus Bio-Medico, Via Álvaro del Portillo, 21, 00128 Rome, Italy

**Keywords:** northeast Brazil, DENV, Dengue fever, genomic surveillance, phylodynamics

## Abstract

Dengue fever is among the most significant public health concerns in Brazil. To date, the highest number of Dengue notifications in the Americas has been reported in Brazil, with cases accounting for a total number of 3,418,796 reported cases as of mid-December 2022. Furthermore, the northeastern region of Brazil registered the second-highest incidence of Dengue fever in 2022. Due to the alarming epidemiological scenario, in this study, we used a combination of portable whole-genome sequencing, phylodynamic, and epidemiological analyses to reveal a novel DENV-1 genotype V clade and the persistence of DENV-2 genotype III in the region. We further report the presence of non-synonymous mutations associated with non-structural domains, especially the NS2A (non-structural protein 2A), as well as describe synonymous mutations in envelope and membrane proteins, distributed differently between clades. However, the absence of clinical data at the time of collection and notification, as well as the impossibility of monitoring patients in order to observe worsening or death, restricts our possibility of correlating mutational findings with possible clinical prognoses. Together, these results reinforce the crucial role of genomic surveillance to follow the evolution of circulating DENV strains and understand their spread across the region through inter-regional importation events, likely mediated by human mobility, and also the possible impacts on public health and outbreak management.

## 1. Introduction

Dengue virus (DENV) is an arthropod-borne pathogen member of the *Flaviviridae* family that spreads through the bite of infected mosquitoes, particularly those belonging to the genus Aedes [1]. DENV is endemic in most tropical and subtropical areas of the globe, putting ~50% of the global population (nearly 4 billion people) at risk of infection and leading to an alarming number of Dengue fever outbreaks [2,3]. Dengue fever is a complex disease characterized by an extensive spectrum of symptoms ranging from asymptomatic cases to life-threatening hemorrhagic pathology [2].

This pathogen is antigenically classified into four serotypes (DENV-1–4), presenting remarkable genomic dissimilarities and distinct spatial–temporal distributions [4]. To date, 19 different genotypes have been reported for the Dengue virus [5]. Even though heterotypic serotype infections are associated with a high risk for severe clinical outcomes, there is a lack of information regarding the impact of the virus genotype on Dengue pathogenesis [6].

DENV was eradicated in several regions of South America during the mid-twentieth century [7]. However, the virus was identified again in Brazil in the early 1980s, causing an outbreak in the Roraima state, in the northern region [8]. Since its reintroduction, Dengue fever has represented a significant public health burden, contributing significantly to loss of life and affecting numerous people annually, which recently appeared to be associated with a substantial increase in severe cases [9,10,11,12,13,14,15,16,17]. Additionally, in recent years, DENV-1 and DENV-2 have become predominant in Brazil [16,17].

Between 2017 and 2022, Brazil accounted for a total number of 3,418,796 reported cases of Dengue fever [18]. Additionally, between 2018 and 2019, Brazil observed a significant 5-fold increase in warning sign cases and a 4-fold increase in the incidence of severe cases and deaths, with northeast Brazil accounting for the second-highest incidence of Dengue fever by the end of 2022 [13,15,16,17,19].

Due to the alarming epidemiological scenario, promoting the genomic surveillance of circulating viral strains appears to be pivotal to anticipate possible impacts on public health and guide outbreak response [6,12,20]. Thus, to provide more insights regarding the molecular diversity of Dengue virus in Brazil, in this study, we performed nanopore sequencing to generate 19 novel genome sequences of DENV-1 and DENV-2 sampled in 2019 from infected patients residing in the city of Feira de Santana, state of Bahia, northeast Brazil.

## 2. Materials and Methods

### Diagnostic and Library Preparation Procedures

Serum samples retrieved from patients presenting symptoms compatible with arboviral infection were collected by the Health Secretary of Feira de Santana-BA and transported to the Instituto Gonçalo Moniz (Fiocruz) for molecular diagnosis. Samples were submitted first to nucleic acid extraction and purification using the Reliaprep Viral TNA kit (Promega) and subsequently to molecular screening using a multiplex RT-qPCR assay to detect Zika, Dengue and Chikungunya viruses (Kit Molecular-ZDC, Bio-Manguinhos). Based on the RT-qPCR cycle threshold (Ct) levels, 17 DENV-1 and 2 DENV-2-positive samples were submitted to genomic sequencing at the Gonçalo Moniz Central Public Health Laboratory (LACEN-BA) in the state of Bahia. cDNA synthesis was performed using the SuperScript IV Reverse Transcriptase kit (Invitrogen) and subjected to sequencing multiplex PCR (35 cycles) as previously described [12]. DNA library preparation was conducted using the Ligation Sequencing kit (Oxford Nanopore Technologies) and Native Barcoding Expansion 1-96 kit (Oxford Nanopore Technologies), following the reaction conditions as previously described in [12]. Sequencing was performed up to 24 h on a MinION device, and the final consensus sequences were obtained using the Genome Detective software [21]. Genotype assignment was performed applying the phylogenetic arbovirus subtyping tool, available at http://genomedetective.com/app/typingtool/dengue.

To investigate the evolution and population dynamics of DENV-1–2 in the state of Bahia, the DENV-1 (*n* = 17) and DENV-2 (*n* = 2) complete genome sequences generated in this study were combined with globally sampled and publicly available complete genome sequences from DENV-1 genotype V (DENV-1–V = 487) and DENV-2 genotype III (DENV-2-III = 620) that were retrieved from NCBI up to December 2022. Sequences without a sampling date and location were excluded, as were sequences covering less than 50% of the viral genome.

All sequences were aligned using MAFFT [22] and manually edited to remove artifacts (insertions, deletions, and gaps) using Aliview [23]. The maximum likelihood (ML) phylogenetic trees were estimated using IQ-TREE2 [24], under the GTR nucleotide substitution model, which was inferred as the best-fit model by the ModelFinder application implemented in IQ-TREE2 [24]. The robustness of the tree topology was determined using 1000 bootstrap replicates. The presence of a temporal signal was evaluated in TempEst [25] and time-scaled phylogenetic trees were inferred using BEAST package [26]. We employed a stringent model selection analysis using both path sampling (PS) and stepping stone (SS) procedures to estimate the most appropriate molecular clock model for the Bayesian phylogenetic analysis [26]. The uncorrelated relaxed molecular clock model was chosen as indicated by estimating marginal likelihoods, also employing the codon-based SRD06 model of nucleotide substitution and the nonparametric Bayesian Skyline coalescent model.

## 3. Results

To investigate the circulation of DENV-1 and DENV-2 in northeast Brazil, we obtained *n* = 17 (DENV-1) and *n* = 2 (DENV-2) new genomes isolated from positive patients using nanopore sequencing. The 19 novel DENV genome sequences had a mean genome coverage of 98.0% (coverage range 97.7–99.6%), with a mean depth of >1000× for all samples. All sequenced samples were collected between March and May 2019 and made available online at GenBank (accession codes: OQ078871-OQ078889) (details in Table 1).

**Figure 1:**
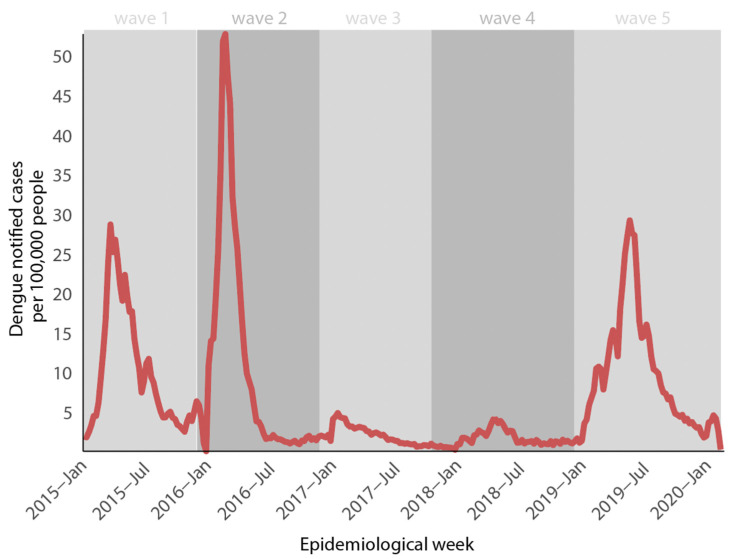
Epidemiological curve of Dengue incidence in the period 2015–2020 in Bahia state, northeast Brazil. Numbers are expressed by the total of DENV reported cases per 100,000 inhabitants reported by epidemiologic week.

This study investigated the molecular diversity of DENV in the city of Feira de Santana, an important metropolitan region and a highway hub located in the state of Bahia, northeast Brazil. All samples were collected during a Dengue epidemic in 2019. Figure 1 shows the DENV weekly cases normalized per 100 K individuals reported between 2015 and 2020 (until epidemiological week–EW 06) in the state of Bahia. Three DENV epidemic waves were found (2015, 2016, and 2019). Before 2015, the epidemiological picture of Dengue virus in Feira de Santana was dominated by DENV-1 (2010–2011) and DENV-4 (2012–2014). DENV-1 was the most common serotype in the municipality of Feira de Santana during the first wave in 2015. However, due to a lack of serotype information post-diagnosis methods, we were unable to determine the most relevant serotype during the second wave. Even though a decline in cases was observed in 2017 and 2018. This pattern has already been observed in other research and can be attributed to a progressive accumulation of herd immunity [12,20] or changes in vector competence (Figure 1). (Data received from the Ministry of Health’s platform—Management of Laboratory Environment (GAL), graciously donated by Professor Gonçalo Moniz of the Central Public Health Laboratory—LACEN-BA.)

**Figure 2:**
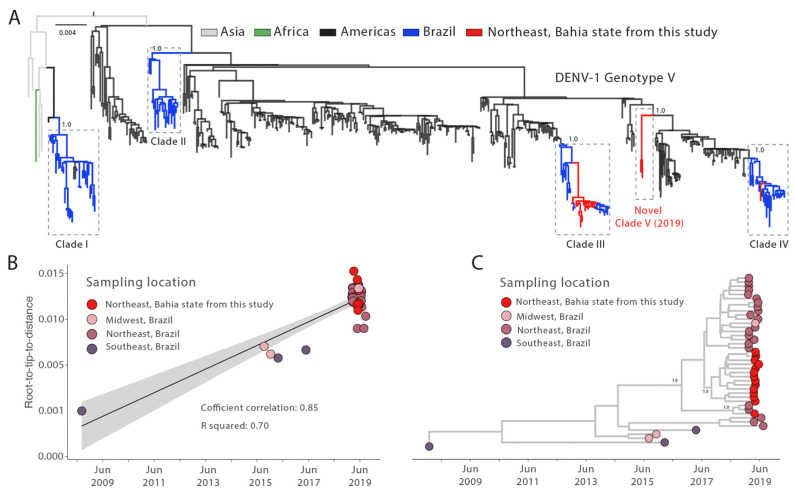
Genomic monitoring of DENV-1 genotype V in Bahia state, northeast Brazil. (**A**) Maximum likelihood (ML) phylogenetic analysis of 17 complete genome sequences from DENV-1 generated in this study plus *n* = 487 available sequences from GenBank. The scale bar is in units of nucleotide substitutions per site (s/s) and the tree is mid-point rooted. Colors represent different sampling locations. (**B**) Regression analysis of the root-to-tip divergence against the sampling date for the DENV-1 genotype V dataset using the statistical approach implemented in TempEst [25]. (**C**) Time-scaled maximum clade credibility phylogeny of Dengue-1 genotype V (DENV-1V) clade of two genomes isolated in Bahia state, northeast Brazil, plus the fourteen new genomes generated in this study and (*n* = 28) Brazilian reference strains from the distinct region. Tips are colored according to the Brazilian region. Values around key nodes represent posterior probability support.

The DENV typing tool classified all DENV-1 genomes generated here as genotype V and DENV-2 genomes as genotype III (Table 1). Our phylogenetic reconstruction revealed that the newly DENV-1 genotype V sequences were placed in three different clades (including an emerging one, hereafter identified as clade V), suggesting that at least three independent introduction events occurred in the state of Bahia in 2019 (Figure 2A). Most of the new generated genomes were closely related to sequences placed in the previously described clade III. Otherwise, a reduced number of sequences was found related to clades IV and V (one and two new genomes, respectively).

In order to investigate the possibility of recombination, we conducted a screening of the sequences using RDP (recombination detection program) to identify any evidence of a recombination pattern. For this analysis, we used the RefSeqs for DENV-1 and DENV-2. Our results indicate that there was no evidence of recombination between the sequences obtained for DENV-1V and DENV-2III in this study.

To investigate the dispersion dynamics of the main clade identified in the state of Bahia, (clade 3, Figure 2A), we built a subset that includes all sequences (*n* = 42) which belong to this clade and submit it to phylodynamic inference. A regression of genetic divergence from root to tip against sampling dates confirmed sufficient temporal signal (coefficient correlation = 0.85, r^2^ = 0.70 Figure 2B). The time-scaled tree revealed that all DENV-1V clade III genomes generated here grouped together with other genomes isolated in Brazilian northeast states, showing the importance of the regional circulation (Figure 2C). Most of them cluster in a highly supported monophyletic subclade for which the time of the most recent common ancestor (TMRCA) was approximately in October 2017 (95%HPD: mid-January 2017 to late May 2018); however, a single genome clustered together with genomes from other northeastern states. Basally in DENV-1V clade III, we can observe some sequences from southeast Brazil, which could suggest an origin of this clade. However, the lack of DENV genomes from before 2018/2019 make more detailed analysis and conclusions difficult.

In addition, we described 28 point mutations among clades I (*n* = 3), IV (*n* = 3) and V (*n* = 22), 21 of which are unique to the novel clade V. Most mutations were associated with amino acid substitutions in non-structural proteins (NS1, NS2A, NS2B, NS3, NS4A, and NS5), Pr (protease), envelope, and RdRp (RNA-dependent RNA polymerase) proteins. As the RdRp and non-structural proteins play a crucial role in viral genome replication, additional research is required to elucidate the potential impact of these mutations on structure and function, and thus on viral pathogenesis (neurovirulence and/or neuroinvasiveness) and fitness (Table 2).

**Figure 3:**
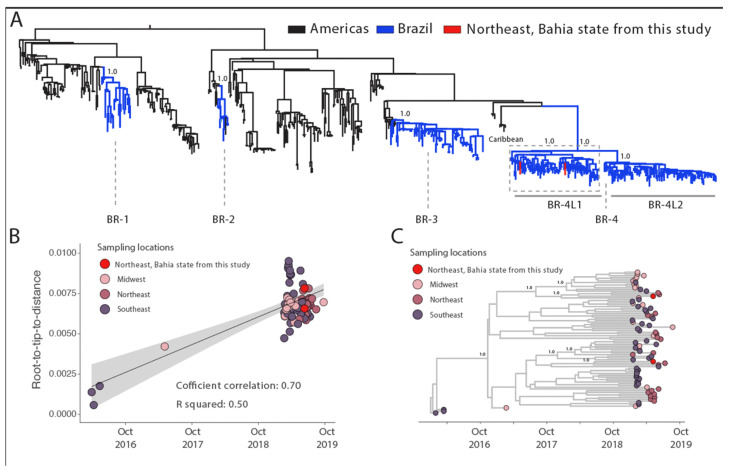
Genomic monitoring of DENV-2 genotype III in Bahia state, northeast Brazil. (**A**) Maximum likelihood (ML) phylogenetic analysis of 2 near-complete genome sequences from DENV-2 generated in this study plus 620 available sequences from GenBank. The scale bar is in units of nucleotide substitutions per site (s/s) and the tree is mid-point rooted. Colors represent different sampling locations. (**B**) Regression analysis of the root-to-tip divergence against the sampling date for the DENV-2 genotype III dataset using the statistical approach implemented in TempEst [25]. (**C**) Time-scaled maximum clade credibility phylogeny of Dengue-2 genotype III (DENV-2III) genomes isolated in Bahia state, northeast Brazil, including the two new genomes generated in this study plus Brazilian reference strains from distinct mesoregions. Tips are colored according to the Brazilian localities. Values around key nodes represent posterior probability support.

To explore the dynamic of DENV-2, we combined our two new sequences to 620 DENV-2 genotype III (DENV-2-III) genomes available on GenBank. Our phylogenetic reconstruction revealed that the novel isolates were closely related to the recently described BR4 lineage 1 (BR4-L1) clade [12] (Figure 3A).

To obtain more insight into the transmission dynamics of the BR4-L1 clade, we then submitted it to phylodynamic inference. A regression of genetic divergence from root to tip against sampling dates confirmed sufficient temporal signal in this subset (coefficient correlation = 0.70, r^2^ = 0.50 for both serotypes Figure 3B).

Furthermore, our maximum clade credibility (MCC) tree (Figure 3C) in line with a previous study [6] revealed the persistence of this viral strain in northeast Brazil, including the state of Bahia and suggested that multiple introduction events occurred through time in that state. The most recent common ancestor of our first isolate dated back to late March 2018 (95%HPD: mid-December 2017 to mid-September 2018) and for the second DENV-2III sequence back to late June 2018 (95%HPD: mid-January 2018 to mid-October 2018) (Figure 3C). The DENV-2-III BR4-L1 clade genomes generated in this study clustered closely with other sequences from northeast Brazil, mirroring what was observed for DENV-1–V clade. However, distinctly from DENV-1–V, clade 2 that was mainly composed by sequences from northeast Brazil, DENV-2-III BR4-L1 was dominated by genomes from southeast and mid-west regions that were evenly distributed across the tree. Further analysis including more genome sequences will be required to reconstruct the ancestral nodes states and the transmission patterns within different Brazilian regions.

## 4. Discussion

In this study, using nanopore sequencing, we generated 17 new genomes of DENV-1 genotype V and 2 of DENV-2 genotype III sampled in 2019 from infected patients residing in the city of Feira de Santana, state of Bahia. Dengue has been a significant public health problem since its reintroduction in the 1980s in Brazil. To date, almost 22,000,000 cases have been reported in the country [18], with the Bahia state reporting the second highest number of reported cases [16,27]. After two years of mild outbreaks, the year 2019 featured the return of a big Dengue epidemic in the state of Bahia. Studies have shown that the reduced number of reported Dengue cases in 2017 and 2018 was a direct consequence of a transient acquired herd immunity, due to the post-Zika (ZIKV) epidemic cross-immunity, as well as the likely changes in the vector capacity and vector control activities implemented by public health agents [28,29,30].

All newly generated DENV-1 genotype V genome sequences were arranged in three distinct clades (III, IV and V). This phylogenetic pattern suggested that between 2015 and 2019, DENV-1 circulated in different Brazilian regions with a succession of different viral clades [6,28,29,31,32,33,34,35]. Additionally, we report the circulation of an emerging clade in the country, herein labeled as clade V. This clade represents a new DENV-1 genotype V lineage introduction in Brazil, with the ancestor positioned among sequences from other South American countries, mainly Argentina and Venezuela. Even though our data reinforce evidence of a new introduction of DENV-1V, these two clustered sequences (clade V) can be inserted in a major clade. However, due to the limited number of DENV sequences generated in South America, the capacity to reconstruct the origins of this clade is limited. In addition, we identified 22 single nucleotide variants within the novel clade V, of which 19 led to amino acid substitutions. Additional research is required to determine the effect of these variants on the structure and function of the associated proteins, as well as their potential role in viral pathogenesis and fitness.

From our dated phylogeny we estimate the tMRCA of the new isolates belonging to DENV-1V clade 3 in Bahia to early October 2017 (95%HPD: mid-January 2017 to late May 2018). These large intervals likely reflect the lack of genomic data during this period, reinforcing the need to strengthen a more effective genomic surveillance program within the country.

The DENV-2 genotype III genome sequences obtained in this study belong to the recently described BR4 lineage one clade, which has already been described as the prevalent strain circulating in Brazil during past epidemics [12,20,31]. Our results further suggest that multiple introductions of this viral strain occurred in the state of Bahia. From our tMRCAs we estimated that the first introduction occurred in Bahia between late March 2018 (95%HPD: mid-December 2017 to mid-September 2018) and the second one in late June 2018 (95%HPD: mid-January 2018 to mid-October 2018), which in turn appear to be mediated by the Midwest and the Southeast regions, respectively, as previously reported in [12].

The assembly of the Dengue virus involves multiple steps, including cleavage of the viral polyprotein into three structural proteins, packaging of the viral RNA, and incorporation of the prM (pre-membrane) and E proteins into virions. However, the molecular mechanisms underlying these events are not fully understood, and mutations associated with capsid and membrane proteins have been reported to play a crucial role in flavivirus virulence [32,33,34]. In this study, we report non-synonymous mutations associated with non-structural domains, especially the NS2A protein, and synonymous mutations in envelope and membrane proteins, distributed differently between clades.

The NS2A protein is responsible for coordinating the organization of the nucleocapsid and the formation of the mature virus [34,35]. Mutations that change the amino acid sequence of NS2A can lead to the structural disorder of the virus, which may contribute to a higher-than-expected pattern of virus–cell interaction. This phenomenon is similar to when changes in the capsid are observed. These modifications can lead to increased virulence and quicker virus replication, directly reducing the action time of the host’s immune system [33,35,36].

In addition to providing genomic information, the presence of mutations in positions relevant to viral assembly and replicative processes can affect the dynamics of infection or pathogenicity. For example, disorder at the Pr/M has been associated with the morbidity of DENV-2, while disorder at C is associated with rapid replication and mortality rates. However, the absence of clinical data at the time of collection and notification limits our ability to correlate mutational findings with clinical outcomes [35].

To better describe the impact of the mutational constellation described between clades, additional analyses such as isolation and infectivity assays would serve as complementary tools. Overall, these findings highlight the importance of understanding the molecular mechanisms underlying viral assembly and replicative processes, and the potential impact of mutations on viral pathogenicity.

As large-scale use of a vaccine against Dengue virus becomes increasingly imminent, it is vital to provide genomic data on viral diversity in a given locality, as well as to describe its spatial distribution and identify risk areas. Such information is essential for decision-making in public health, and the identification of risk areas, as well as vulnerable groups, is critical for the development of preventive measures and awareness campaigns. Collaboration between scientific researchers and public health laboratories in expanding testing and population serological assays can help to identify individuals suitable for vaccination, thereby reducing the risk of illness in the event of post-vaccination primary infection.

In summary, our results revealed a surprisingly high genetic diversity of DENV in a single outbreak sampling in the city of Feira de Santana, state of Bahia, Brazil. The 19 DENV genomes we generated here were clustered in three different clades of DENV-1 genotype V and in two locations within the widespread clade BR4-L1 of DENV-2 genotype III. These findings show a complex dynamic of DENV viral lineages circulation within Brazil and among South American countries. Together, these results highlight the crucial role of genomic surveillance and show that genomic data can be employed to assist public health laboratories in monitoring the diversity of circulating mosquito-borne viruses which in turn will be pivotal to adopting effective control measures and managing future epidemics.

## Figures and Tables

**Table 1:** Epidemiological data of the 19 DENV-1–2 samples sequenced as part of this study.

ID	Virus Type	Genotype	Country	Isolate	Sample Type	Collection Date	Accession Number
NIH-05_NB14_BA	DENV-1	Genotype V	Brazil: Bahia	Human	Serum	12-Apr-2019	OQ078871
NIH-06_NB15_BA	DENV-1	Genotype V	Brazil: Bahia	Human	Serum	21-Mar-2019	OQ078872
NIH-07_NB16_BA	DENV-1	Genotype V	Brazil: Bahia	Human	Serum	21-Mar-2019	OQ078873
NIH-08_NB17_BA	DENV-1	Genotype V	Brazil: Bahia	Human	Serum	23-Mar-2019	OQ078874
NIH-13_NB18_BA	DENV-1	Genotype V	Brazil: Bahia	Human	Serum	30-Mar-2019	OQ078875
NIH-14_NB19_BA	DENV-1	Genotype V	Brazil: Bahia	Human	Serum	02-Apr-2019	OQ078876
NIH-15_NB20_BA	DENV-1	Genotype V	Brazil: Bahia	Human	Serum	12-Apr-2019	OQ078877
NIH-16_NB21_BA	DENV-1	Genotype V	Brazil: Bahia	Human	Serum	09-Apr-2019	OQ078878
NIH-18_NB22_BA	DENV-1	Genotype V	Brazil: Bahia	Human	Serum	10-Apr-2019	OQ078879
NIH-19_NB23_BA	DENV-1	Genotype V	Brazil: Bahia	Human	Serum	09-Apr-2019	OQ078880
NIH-20_NB24_BA	DENV-1	Genotype V	Brazil: Bahia	Human	Serum	12-Apr-2019	OQ078881
NIH-21_NB25_BA	DENV-1	Genotype V	Brazil: Bahia	Human	Serum	24-Mar-2019	OQ078882
NIH-22_NB26_BA	DENV-1	Genotype V	Brazil: Bahia	Human	Serum	21-Mar-2019	OQ078883
NIH-23_NB27_BA	DENV-1	Genotype V	Brazil: Bahia	Human	Serum	16-Apr-2019	OQ078884
NIH-24_NB28_BA	DENV-1	Genotype V	Brazil: Bahia	Human	Serum	11-Apr-2019	OQ078885
NIH-25_NB29_BA	DENV-1	Genotype V	Brazil: Bahia	Human	Serum	30-May-2019	OQ078886
NIH-27_NB30_BA	DENV-1	Genotype V	Brazil: Bahia	Human	Serum	23-May-2019	OQ078887
NIH-26_NB39_BA	DENV-2	Genotype III	Brazil: Bahia	Human	Serum	08-May-2019	OQ078888
NIH-28_NB40_BA	DENV-2	Genotype III	Brazil: Bahia	Human	Serum	08-May-2019	OQ078889

**Table 2:** Profile of single-nucleotides mutations found for DENV-1 genotype I, IV and V clades in isolates generated in this study.

Position	Ref	Alt	Mutation	aa Change	Clade	Genomic Region
253	C	T	S	I-I	I	anchored capsid protein ancC
1100	C	T	S	T-T	I	envelope protein E
1309	G	A	S	T-T	I	envelope protein E
859	A	G	S	S-S	IV	membrane glycoprotein M
1246	T	C	S	W-W	IV	envelope protein E
8533	C	G	S	G-G	IV	RNA-dependent RNA polymerase NS5
703	C	G	NS	Q-E	V	protein pr
8533	C	G	NS	Q-E	V	RNA-dependent RNA polymerase NS5
2273	T	C	NS	F-S	V	envelope protein E
3950	T	C	NS	F-S	V	non-structural protein NS2A
8150	T	C	NS	F-S	V	RNA-dependent RNA polymerase NS5
3090	T	C	S	H-H	V	non-structural protein NS1
3227	T	C	NS	L-P	V	non-structural protein NS1
3790	A	G	NS	S-D	V	non-structural protein NS2A
3791	G	A	NS	S-D	V	non-structural protein NS2A
3806	G	A	NS	G-D	V	non-structural protein NS2A
3914	G	A	NS	G-D	V	non-structural protein NS2A
3883	A	G	NS	T-V	V	non-structural protein NS2A
3884	C	T	NS	T-V	V	non-structural protein NS2A
3892	T	A	NS	F-I	V	non-structural protein NS2A
3959	C	G	NS	A-G	V	non-structural protein NS2A
3994	A	T	NS	N-Y	V	non-structural protein NS2A
7912	A	T	NS	N-Y	V	RNA-dependent RNA polymerase NS5
4316	G	C	NS	R-T	V	non-structural protein NS2B
4597	C	G	NS	P-A	V	non-structural protein NS3
6643	T	G	NS	C-G	V	non-structural protein NS4A
6879	C	T	S	C-C	V	non-structural protein NS4B
7977	C	T	S	Y-Y	V	RNA-dependent RNA polymerase NS5

Nucleotide position reported from alignment with reference sequence RefSeqNC001477 available in the NCBI RefSeq database. S: synonymous mutation; NS: non-synonymous mutations.

## Data Availability

Newly generated DENV sequences have been deposited in GenBank under accession numbers OQ078871-OQ078889.
